# A crossroad for validating digital tools in schizophrenia and mental health

**DOI:** 10.1038/s41537-018-0048-6

**Published:** 2018-04-06

**Authors:** John Torous, Patrick Staples, Ian Barnett, Jukka-Pekka Onnela, Matcheri Keshavan

**Affiliations:** 1 Beth Israel Deaconess Medical Center, Harvard Medical School, Boston, MA USA; 2 Department of Biostatistics, Harvard T.H. Chan School of Public Health, Boston, MA USA; 3 Department of Biostatistics and Epidemiology, University of Pennsylvania, Philadelphia, PA USA

Schizophrenia remains one of the most devastating chronic illnesses,^1^ impacting nearly 1.5% of the global population^2^ and creating an economic burden of up to 1.65% gross domestic product.^3^ It is not surprising that digital tools for schizophrenia, often smartphone-based software in the form of apps, have received so much recent attention and enthusiasm. Digital phenotyping holds tremendous potential in elucidating the complex heterogeneity of what we call schizophrenia and would therefore advance research. On a clinical front, smartphone data may become increasingly valuable in monitoring course and treatment response given the fact that these patients frequently have difficulties in adherence with clinical visits, and are often poor historians. However, the power of this paradigm is currently fueled more by the increasing ubiquity of technology than breakthroughs in clinical science. The accessibility and affordability of digital care derives from increasing global ownership of smartphones: it is estimated that six billion smartphones will be in circulation worldwide by 2020.^4^ As devices and sensors become ever cheaper and more sophisticated, the ability to capture a plethora of relevant data and deliver a myriad of content via network connectivity will continue to fuel the potential of digital approaches in mental health. As validation and reproducibility lags behind enthusiasm and availability, the potential clinical impact of these digital tools is at a crossroads.

How might this new approach advance clinical care? Affordable and accurate diagnostics from smartphones paired with on-demand or automatically deployed interventions enables unprecedented access to mental health services. Apps today are designed to perform a wide range of healthcare tasks ranging from telehealth to medication tracking. In addition, new platforms are currently being developed to measure novel behavioral and physiological markers using passive long-term smartphone data, enabling objective measurement without burden for patients and healthcare providers. Clinically relevant and passively collected smartphone data comprises a wide range of sensors, including accelerometer data to estimate activity, anonymized call/text log information to estimate sociability, and screen touch data to estimate cognition. Passive measurement might also be able to distinguish disease subtypes to help better classify psychotic illnesses, similar to recent research using genetic, physiological, and cognitive markers.^5^

The considerable potential impact of these tools is paralleled by substantial new challenges. Formidable analytical complexities accompany all data-driven approaches, including clinical inference using passive smartphone data. Missing data, the high-dimensional and temporally dense nature of the collected data, habits of smartphone use, quality of user experience with the app, and the quality of the software implementation may all act as confounders to underlying clinical disease state. Estimates of clinical accuracy and efficacy of these devices remains broadly understudied. Daunting implementation challenges also accompany this smartphone-based work: simple questions such as which patients are comfortable with smartphone monitoring, how long should it be used for, how information should be shared with patients and psychiatrists all remain largely unknown. Ethical questions also remain with respect to appropriate storage, access, and usage protocols for this highly personal data.^6^

Ignoring these challenges and questions would be both a scientific mistake and also a missed opportunity for clinical care. Recall the humorous 2009 case report of the dead North Atlantic Salmon, who was asked to detect emotions in photos during a fMRI task, resulting in the “finding” of correlated neural activity—due to failing to control for multiple comparisons.^7^ Online analyses of digital phenotyping data performed on say, a daily basis, are faced with the same challenge of correcting for multiple comparisons. The more recent discovery of widespread statistical software issues in thousands of fMRI research protocols underscores how simple mistakes can be amplified with digital tools.^8^ The promise of fMRI is owed to its high-resolution detail, which is directly tied to data complexity and the peril of inappropriate statistical inference. Digital phenotyping^9^ data, which is in situ, multi-sensor, partially observed, and longitudinal, brings even more complexity to bear, and deserves proportionate circumspection.

To meet these challenges and to hone this approach into clinically useful tools, the development of research platforms must be accompanied with empirical research on the properties of the data it collects, called metadata. A simple example of metadata is the time it may take you respond to a smartphone query, instead of the response to the query itself. Studying metadata is useful for two reasons. First, understanding the limitations and biases of our tools used to draw clinical inferences will improve their specificity and clarify where they can be beneficially used. Second, metadata itself might offer novel insights about patient behavior and especially cognition that is not available using traditional metrics or evaluations. Recent research from our group^10^ suggests that properties of smartphone data may be more complex than often portrayed in the popular press, which holds relevance for clinical use. We show a correlation in the outcomes between metadata such as accelerometer coverage, GPS coverage, and survey completion timings, and future responses to questions about mood, anxiety, and psychotic symptoms. This might be evidence that these measures might help predict disease progression. We also find that some of these measures differ by operating system (iOS vs. Android), potentially indicating confounding by operating system or other variables, such as socioeconomic status.

In addition to understanding metadata, the development of digital phenotyping tools will benefit from other considerations. Data standards and extensive testing may take significant forethought and precious resources, but such care typically helps the success of deployment in the long run. Without standards in collecting, processing, and reporting for digital phenotyping data, the end result might be a continuation of the pilot studies we currently see, which are expensive and their results are often left unreplicated. This pales in comparison to the value of high-throughput, well-coordinated, multi-site research efforts seen in fields such as genetics and molecular life sciences. As digital tools are built with the apparent goal of providing and complementing current care, choices made now will reflect on the replicability and effectiveness of these tools once widely deployed.

Although the digital phenotyping approach requires extensive validation, embracing the complexity of tools now, including a thorough understanding of clinical metadata, will enable new ways to understand mental illnesses and deliver personalized medicine for schizophrenia and related disorders.^11^ At the crossroads of validation and reproducibility versus enthusiasm and availability lies the challenge to bridge both sides and realize the full potential of these new digital tools.

The crossroads of the field presents a choice of path along which this important field will develop.
